# Drivers of purchase decisions for cannabis products among consumers in a legalized market: a qualitative study

**DOI:** 10.1186/s12889-021-12399-9

**Published:** 2022-02-21

**Authors:** Jennifer Donnan, Omar Shogan, Lisa Bishop, Maisam Najafizada

**Affiliations:** 1 School of Pharmacy, Memorial University, St. John’s, Newfoundland and Labrador Canada; 2 Faculty of Medicine, Memorial University, St. John’s, Newfoundland and Labrador Canada

**Keywords:** Cannabis, Marijuana, Purchase decisions, Attributes of choice

## Abstract

**Background:**

Cannabis was legalized in Canada for non-medical use in 2018. The goal of legalization was to improve health and safety by creating access to regulated products, with accurate product labels and warnings and no risk of contamination. However, more than 2 years post-legalization, a large proportion of purchases are still suspected to be through unlicensed retailers. This study sought to identify the factors that influenced the purchase decisions of cannabis consumers in Newfoundland and Labrador (NL).

**Methods:**

Semi-structured focus groups and interviews were conducted in NL with individuals who were > 19 and had purchased cannabis within the last 12 months. All sessions were conducted virtually, audio-recorded, and transcribed. A thematic analysis was conducted, and two members of the research team coded the data using NVivo. A combination of deductive and inductive coding was carried out, themes from the literature were identified, and new themes from the transcripts were discovered. A final coding template of the data was agreed upon by the team through discussion and consensus.

**Results:**

A total of 23 individuals (30% women) participated, with 83% coming from urban areas. While all cannabis product types were discussed, the conversation naturally focused on dried flower products. Participants discussed a variety of considerations when making purchase decisions categorized around five broad themes: 1) price, 2) quality, 3) packaging and warnings, 4) the source of the cannabis, and 5) social influences. The price difference between licensed and un-licensed sources was commonly discussed as a factor that influenced purchase decisions. Product quality characteristics (e.g. size, color, moisture content) and social influences were also considered in purchase decisions. Participants were generally indifferent to packaging and warning labels but expressed concern about the excessive packaging required for regulated products.

**Conclusion:**

This study explores the many attributes that influence purchase decisions for dried leaf cannabis. Understanding the drivers of purchase decisions can help inform policy reforms to make regulated cannabis products more appealing to consumers. Further research is needed to measure the effect of each attribute on cannabis purchase decisions.

## Background

**Fig. 1:**
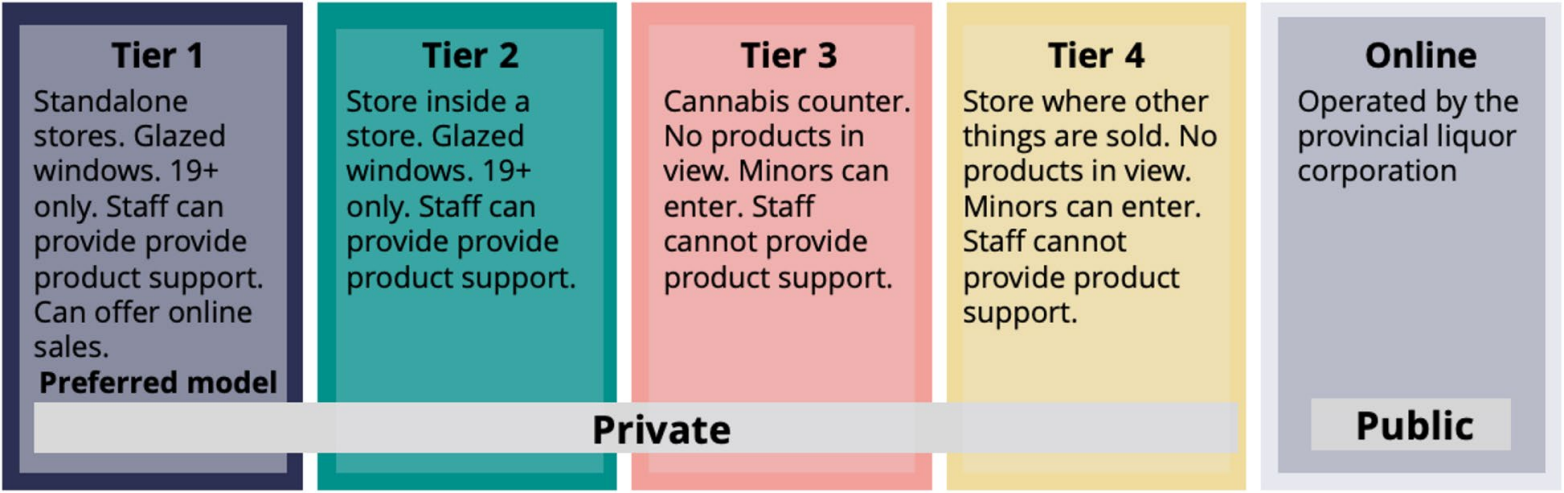
Retail Sales Model in Newfoundland and Labrador

The non-medical use of cannabis was legalized in Canada on October 17, 2018. The federal *Cannabis Act* entails specific regulations; however, each province and territory were responsible for establishing legislation and regulations for certain requirements such as the minimum age of purchase, quantity of possession, licensing the distribution and retail sale (Textbox 1) [1]. Provinces could opt for sales through government run (public) stores or websites, privately run stores or websites, or a hybrid model of public and private sales (Table 1) [2]. Newfoundland and Labrador took a unique approach where they implemented a four-tier private retail model, where different regulations were put in place depending on the physical retail environment (Fig. 1).

**Table:** 

***Textbox 1. Highlights of the Canadian Cannabis Act***	
*Age of consumption:* 18 years and over (though the minimum age can be higher in individual provinces)	
*Quantity:* An individual may only possess 30 grams of cannabis, and each household can grow up to four plants for their own use.	
*Packaging:* Strict rules on branding and advertising. The package must include a health warning and information on the amount of cannabidiol (CBD) and delta-9-tetrahydrocannabinol (Δ-9-THC).	
*Cannabis-infused food and drinks:* Edible cannabis products may only be sold with a maximum THC amount of 10 mg per package.

**Table 1:** Cannabis Retails Sales Models in Canada

Public	Private	Mixed
Prince Edward Island	Manitoba	Newfoundland and Labrador (public online, private in-person sales^a^)
Nova Scotia	Saskatchewan	Ontario (public online, private in-person sales)
New Brunswick	Nunavut	Alberta (public online, private in-person sales)
Quebec		British Columbia (public in-person or online, private in-person stores)
Northwest Territories		Yukon (public online, private in-person sales)

^a^As of 2021 some private stores are permitted to sell through their website

At 2 years after legalization, a significant proportion of cannabis sales still occurred through the Illegal/unlicensed market [3]. Crowdsourced data (anonymously shared information on an internet platform by members of the public) collected by Statistics Canada found that illegal sales made up 41% of the cannabis market as of the end of 2019 [4]. Illegal sources in Canada include unlicensed retailers that are known to many and tolerated by authorities to some extent, such as dispensaries or on-line retailers, as well as acquaintances or drug dealers with potential links to criminal networks. Media reports have noted issues with licensed cannabis sources, including high cost [5–7], poor quality [8, 9], limited supply, distance to licensed stores [5–7], and inconvenient packaging [10]. Crowdsourced data in Canada show that the price per gram of cannabis from a licensed retailer was almost double that sold from unregulated sources [4].

While all these factors likely influence cannabis purchase decisions, limited published research has explored consumer preferences for cannabis products in a post-legalization environment. A review of the literature to identify attributes of choice for cannabis products identified studies that explored consumer preferences (e.g. price, packaging, aroma) but did not capture any studies that sought to identify attributes that influenced choices from the consumer perspective.

Understanding the factors consumers consider when purchasing cannabis will allow policymakers to evaluate the regulations regarding cannabis sales in Canada to ensure that the public can access safe, government-licensed cannabis that also meets consumer needs. Consumer choices can be influenced by the local environment and regulations. While several provinces use a mixed model, Newfoundland and Labrador (NL) is unique in that it adopted a four-tiered private model for in-person sales, with online purchases controlled by the public regulator [11]. Using a qualitative approach, this study explored the factors that influence purchase decisions of cannabis in NL from consumers’ perspectives.

## Methods

A qualitative research design was used as it is considered the most appropriate tool to explore the perspectives of participants [12].

### Participant recruitment

Stratified purposive maximum variation sampling was the first method of recruitment. Participants had to be 19 years of age or older, had to have purchased cannabis in the last 12 months, and resided in NL. Individuals working within the cannabis supply chain were excluded. We attempted to recruit individuals representing various cannabis purchasing patterns, age groups, genders, and geographic regions. Participants were recruited through a variety of mechanisms, including social media campaigns, posters, radio ads, and media interviews, and they were offered a $20 electronic gift card in recognition of their time.

### Data collection

Semi-structured focus groups and interviews were conducted between July 2020 and February 2021. Participants selected a focus group time that suited their personal schedule. While focus groups were preferred to allow for discussion between participants and the building on of ideas, interviews were offered if the participant requested to remain anonymous or if it was required for scheduling purposes. Public health restrictions due to COVID-19 prevented any in-person sessions, however the use of a virtual platform (Zoom) allowed for recruitment across the entire province, which would not have been feasible for in-person sessions. This was more convenient for participants, taking less time out of their day and allowing them to choose from more session times. Due to the challenges of having open discussions through a virtual platform, focus group size was set to a maximum of five participants, which allowed for all participants more opportunity to share their thoughts and opinions. In advance of the session, participants were asked to complete a demographic questionnaire over the phone. During sessions, participants were asked probing questions from a list of open-ended questions with additional probing questions added or modified accordingly as the sessions progressed. Two facilitators ran each session (JD, OS). Sessions lasted between approximately 40 and 70 min, were audio recorded through Zoom, and were transcribed for analysis using NVivo transcription software. Each transcript was then manually verified by listening to the audio recordings. Data were collected until saturation was met.

### Analysis

A thematic analysis was conducted using NVivo. A combination of deductive and inductive line-by-line coding was carried out. Some themes had been pre-identified through literature and media articles (e.g. price, quality, product recommendations), and new themes were identified as they emerged from the transcripts. A sample of transcripts were initially coded, and two team members (JD, OS) met to discuss and organize data into themes and sub-themes. This coding template was then applied to the remaining transcripts and discrepancies were resolved by engaging a third team member. Constant comparison was used to explore relationships between and across thematic codes and between coders to maintain consistency. Once all data was coded, all authors met to finalize the thematic groupings through discussion and consensus. Contradictory statements were sought through the coding process, and balanced description of the findings are presented. The results are reported thematically, with some quotes edited for clarity.

### Research team and reflexivity

The team was comprised of a gender-balanced, culturally diverse group including healthcare researchers in the disciplines of pharmacy and medicine who had experience in qualitative data collection and analysis and a pharmacy trainee (OS). The interview team included a man and woman interviewer. Throughout the study we were aware of our health care professional backgrounds and made every effort to ensure that this did not impact our data collection and interpretation processes. Additionally, we made sure that we maintained a neutral perspective and discussions were not guided to heavily toward medical uses of cannabis. We were aware that healthcare professionals can be considered to hold a social position of power compared to the general public. The interviewers consisted of an experienced researcher and a trainee to mitigate potential issues of power.

### Ethics

This study was approved by Memorial University’s Interdisciplinary Committee on Ethics in Human Research (ICEHR: Approval #20210143) and conducted in accordance with the Tri-Council Policy Statement. Consent was obtained and participants were informed that confidentiality and anonymity would be maintained by the research team. All participants were asked to respect the confidentiality of other focus group participants. One-on-one interviews were arranged for those who wished to maximize their privacy.

## Results

A total of 23 individuals were included, with 15 participating in focus groups and 8 completing interviews. Among the participants, about a third were women, a range of ages and both rural and urban communities were represented. With respect to cannabis use history, most participants were relatively frequent purchasers, with 74% making one or more purchases a month, and 100% were consumers prior to legalization. However, some indicated they only started consuming cannabis once it was announced that legalization was coming, and they considered themselves newer users. In addition, 87% lived in an area with access to an in-person licenced cannabis retailer. While the most common cannabis product used was dried flower (91%), many participants indicated they purchased and consumed more than one product type, including edibles (61%), oils (40%), vaping liquid (17%), beverages (26%), and others (9%) (Table 2). Participants tended to have experience purchasing cannabis from both licensed and unlicensed sources.

**Table 2:** Participant Characteristics

Variable (***n*** = 23)	n (%)
**Gender**	
Man	16 (69.6)
Woman	7 (30.4)
Other	0 (0)
**Age**	
19–29	7 (30.4)
30–39	6 (26.1)
40–49	8 (34.8)
50–59	1 (4.3)
60 +	1 (4.3)
**Area of residence**	
Urban area with 50,000 or more residents	19 (82.6)
Rural area with less than 50,000 residents	4 (17.4)
**Highest level of education completed**	
High school diploma	2 (8.7)
Some post-secondary	4 (17.4)
Trade/technical/vocational training completed	4 (17.4)
University degree	13 (56.5)
**Employment status**	
Full-time student	2 (8.7)
Unemployed	3 (13.0)
Employed full time	15 (65.2)
Self-employed	2 (8.7)
*No answer*	1 (4.3)
**Household income**	
Less than $25,000	4 (17.4)
$25,000 – $49,999	6 (26.1)
$50,000 – $74,999	3 (13.0)
$75,000 – $99,999	3 (13.0)
$100,000 or more	7 (30.4)

**Fig. 2:**
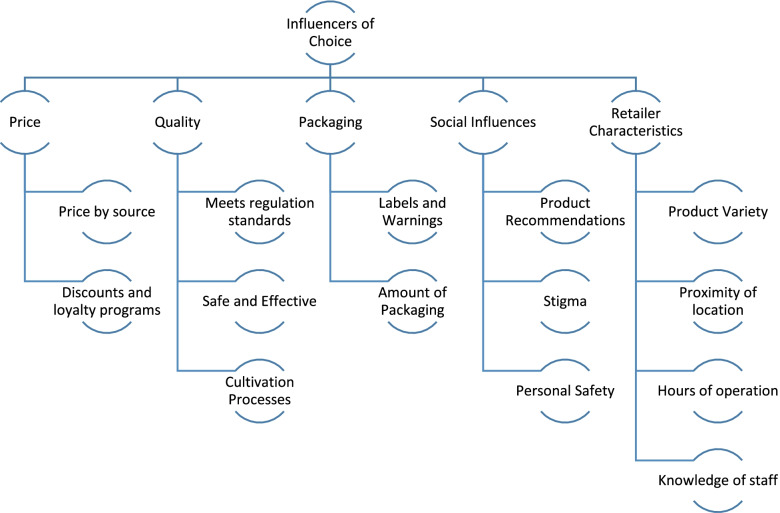
Coding Tree of Identified Themes

Analysis of the transcripts resulted in categorizing the participants’ drivers of purchase decisions into five broad themes: 1) price, 2) quality, 3) packaging, 4) social influences, and 5) retailer characteristics (Fig. 2). The comments raised in focus groups and interviews shared common themes, however interview participants shared their views with more depth given they were not sharing discussion time with others.

### Price

Price was the most commonly discussed concern, as participants frequently spoke of lower prices in the illegal market, the significance of the lower prices for different types of use, and the offering of incentives, loyalties, and discounts in the illegal market.

While there was some variability in the degree of the price differences, most participants noted that legally sourced cannabis was more expensive: *“I don’t feel like going into the stores sometimes it’s so expensive”*. Several people noted that a standard price for a gram of cannabis had been fairly consistent for many years, and once cannabis was legalized the prices of unregulated products dropped. As one participant noted: *“As long as you lower the price, the black market is gonna lower, and you’re just gonna get more money and bang for your dollar, and you have more choice.”* One participant over 60 years of age, who had decades of experience purchasing cannabis, said “*A gram all my life has been 20 dollars and you wouldn’t get off it and nobody would change that”,* and therefore prices in both the licensed and unlicensed market are now much lower than they were ever used to paying. It was also mentioned that prices have gone as low as $3–4 per gram, but the quality of the product was questionable: *“I’ve seen some ridiculously good deals where it’s like, you know, four dollars a gram, three dollars a gram. What’s the quality of it? I don’t know*.” It was described that prices varied slightly by quality, potency, and geographic location. It was also discussed that unlicensed sellers could deliver the product, which was convenient for those who did not have transportation.

This price difference mattered the most to experienced and/or frequent users. Experienced users described that they were already comfortable with purchasing through unlicensed retailers and knew how to source a product they like and feel safe using. Those who frequently used cannabis for either medical or non-medical purposes reported that purchasing through legal stores was unsustainable due to high costs, stating, *“the reason that I stopped purchasing medical marijuana [is] because … I’d go bankrupt”*. In comparison, those who were new or less frequent consumers tended not to be as concerned about the price.

Several participants who were medical users commented that the legalization of non-medical cannabis pushed them to purchase through the unlicensed retailers. While prices between the licensed medical retailers and the unlicensed market were comparable pre-legalization, the price difference post-legalization made it less appealing to continue purchasing from legal sources. As one participant said, “*I was prescribed five grams per day. I would literally have to be homeless to be able to afford to purchase that from a medicinal source*”. It was also a concern that cannabis was not covered by any health insurance plans: *“I have a really good health care plan from my employer, and it’s still not covered through that”.* While some participants had a valid medical reason for using cannabis, they could not get any financial support because of their income level. As explained by one participant:

*“Some of the licensed producers for the medical market do offer things like compassionate pricing and stuff, which can bring them below the recreational market. But that also applies to people who make less than $25,000 a year.”*

Participants also noted that unlicensed retailers offer better sales, loyalty programs and bulk purchase incentives. Customers can get special deals around holidays or even daily or weekly flash deals that promote specific products. As noted by one participant: *“local places that sell online, businesses that sell in the black market, they all have sales for events like Canada Day sales…They will throw in free stuff.”* Many online retailers have point systems that can be used towards discounted or free products or free shipping: *“you get this many points and you get percentages off of future orders and stuff like that.”*

Unlicensed online retailers may offer free gifts with a purchase, such as *“a free gram with your first purchase”*. Purchasing in bulk also allowed for substantial cost savings, which was felt to be worth the large purchase despite potential delays in shipping:

*“a lot of sites, when you get over a certain amount, will give you free shipping and express it. So even with the shipping to Newfoundland being a little delayed, it’s maybe still two to three days to get it, and for the price savings, totally worth it.”*

Some noted that it was not really possible to purchase in bulk at a licensed store because of the limit of 30 g in a single transaction. Some noted that while legal stores offered some specials, they were still not comparable to the discounts offered from the unlicensed retailers.

### Quality

#### Defining quality

A shared understanding of cannabis quality did not exist among the participants. While not mutually exclusive, the participants’ perception of quality was broken down into three main categories regarding the product: 1) meeting federal regulation standards, 2) safe and effective, and 3) cultivated to cater to the user’s experience. Both men and women and those from older and younger demographics fell into each of these categories.

Generally, those people who defined quality as meeting federal regulation standards were individuals who were less experienced with consuming cannabis. They were either new users, returning users since legalization, or infrequent users. They focused on issues such as the accuracy of labels and reducing the risk of contamination from bugs, mites, mold, and other street drugs.

*“But one of the concerns I do have with the black market is that as there’s no regulations. One of the things that spooks me a bit is the fact that I don’t know what, if any, pesticides, insecticides are being used. And I know a huge issue in the black market is when there are mites and bugs that get into the crops, they defecate on the product. And that, of course, is something that you could be inhaling. And I know that the government regulations at least are supposed to help prevent that.”*

These consumers preferred to purchase from licensed retailers to get a quality product.

*“I know if buy a product from [licensed retailer], I know I’m getting good quality. I know it’s regulated. I know if the bottle says they’re in cans of two milligrams, I’m getting two milligrams.”*

Some participants referred to product effectiveness and safety when considering quality. People wanted to know that the product was going to give them a desired effect (e.g., a high, relaxed feeling, better sleep). As one participant said: *“cannabis is a bit more of a means to an end for me. So, quality for me is: Did it get me high, [and] how high did it get me?”*

Participants also wanted to know they would not experience unwanted effects. *“Sativa definitely causes more anxiety…. Indica, I find are more of a sedating effect which has a better effect with anxiety.”*

Some participants noted that certain varieties caused them to feel anxious or sick. These participants talked a lot about trust in their retailer and knowing what was in the product they were purchasing. To get safe products, some consumers bought from licensed retailers, while others felt they could get safe and effective products from unlicensed retailers. From a safety perspective, participants who bought cannabis products from unlicensed markets expressed concern about the potential for pesticide use, improper cultivation, and/or the potential for mold and contamination with unwanted additives.

Finally, more frequent consumer’s generally defined quality based on characteristics that influenced their experience with the product, such as how it was cured and how it looked, smelled, tasted and felt when inhaled. Participants remarked that the curing process greatly influenced the quality, bringing out certain desired flavors while destroying unwanted chemicals and flavors. Participants said that improperly cured cannabis can lead to mold growth if the conditions are too humid. Conversely, improper curing may also leave the cannabis unappealingly dry.

Visually, participants assessed the product moisture content (avoiding anything too dry and brittle), the bud size (smaller and lower-quality buds tend to come from lower on the plant), and the ratio of flower to stems (with a preference for fewer stems). Some also mentioned the trichomes and looking for certain colours that indicated ripeness.

With respect to the smell, taste and feel of the inhaled smoke, participants mentioned that the variety of terpenes in different plant strains could influence the user experience and choice, but there was no specific flavour profile that indicated a higher- or lower-quality product. Most participants described that they did not enjoy a “harsh” feel when they inhaled cannabis; however, there were some who preferred this experience, highlighting different consumer preferences.

*“I look at the trichomes and the trichomes of cannabis; it’s almost like a little bulb, like a little mushroom that grows on top of the calyx or leaf. And that’s what fills up like the cloudy or the amber tone, which gives you your high. So I’d like to know if it’s ripe or not ripe. So when it’s clear, it’s not ripe. I want to make sure the quality I’m getting* [that it] *has been cured and ripened properly. And by having it that way, it allows me to have a smooth smoke and quality that’s going to last a while versus something that’s gonna be airy, not tight, very loose.”*

#### Quality by source

For those that indicated that cannabis quality related to the cultivation process, they suggested that products available from licensed retailers were of lower quality, with very dry leaves, small buds and more stems. It seemed, though, that the products of licenced retailers may be improving. It was noted that many products now contain two-way humidity control packets to keep the product from getting too dry. Some noted, however, that this did not always correct the problem. If the cannabis was not cured properly to begin with, then adding moisture back in did not improve the quality.

People with experience using unlicensed online vendors indicated that the products were of superior quality compared to licensed stores. However, those who ordered cannabis from websites tended to be more experienced users who were comfortable with finding information about the products and placed significant trust in the websites’ product reviews. Participants noted that some online products were not always what they seemed; for example, as one participant said:

*“There are people online who sell what is called ‘washed weed’... It’s a thing where people use it to make things like extracts, but the flower is still intact, but most of the psychoactive effects have been removed from it by a chemical process. So, that’s a thing that happens online that you’ve got to be aware of. You can buy weed online that looks great and smells great but actually doesn’t have any effect at all.”*

Therefore, online shoppers were highly selective of the sources they purchase from. Some participants also highlighted that unlicensed online retailers use an AAAA quality grading system, with AAAA or “quads” being the highest quality, enabling consumers to select any quality grade they like. This grading system does not seem to be used in the licensed market, and participants suggested that the highest-quality cannabis could not be sold in licensed stores because production regulations prevented products of that quality from being produced.

There appeared to be a wide variability in the quality of products purchased from friends or unlicensed community dealers. This variability may stem from the originating source. Participants reported that unlicensed community retailers could get their supply from either growing it themselves, from licensed retailers (and sell it with a mark-up in their community), or from larger unlicensed cultivator/retailers. Therefore, depending on the place of residence and familiarity with cannabis sellers, one could receive products with very different levels of quality.

For those that defined quality based on safety, they agreed that licensed cannabis products were safe, but so were some unlicensed sources. However, it was noted that while there was a lot a variability in the safety of products available from the unlicensed retailers, there were trustworthy sources that offer high-quality, safe products. Participants indicated that reading online reviews was helpful for sourcing trustworthy products. “[I] *talk to people who I know who have ordered from sites. Never just go in blind and order somewhere.”*

### Packaging

Participants were generally indifferent to branding and warning labels, but they were strongly opposed to the excessive packaging that comes with licensed cannabis products. Some commented that the warnings and product information (on the potency, strain, etc.) were important and they wanted to see them. As one person said, *“I prefer to have labels like full labels, I prefer to have full warning signs, and I definitely want to know where it came from… so I can guarantee myself and my friends have a safe* [experience]*.*” While others were indifferent to label characteristics, no one was opposed to the labelling.

Most people who purchased through legal sources commented on the packaging and how it was excessive. As one participant indicated: *“The pre-rolls yea, like you got a box and then you got a tube, and you got the plastic around that tube, and you got to pop that to open.”* They were aware that packages had to be child-safe but felt there could be a better way. Some adults even struggled to get through all the packaging. As one older participant indicated “*they’re a bit hard to get open for some people like myself and I can imagine for someone with bad arthritis*”. Some younger participants also indicated that certain retailers offer a recycling program, which people were pleased with, but it is suspected that most containers do not get recycled.

Some people felt that the packaging also impacted the quality of the product. The containers are larger than they need to be. As one participant said: *“You get a big container… you get a little bit of product in a big old container… I think that maybe adds to the fact that it gets drier quicker because it’s not as much in there.”* Some companies are adding moisture packets or strips to prevent the product from drying out, but customers cannot see the contents before purchasing so it is unclear which companies take extra steps to maintain their product quality.

People who purchased from the unlicensed retailers in the community said that they often received their cannabis in a plastic bag. They liked the minimal packaging, but no product information was included. People who purchase from online unlicensed retailers say that some of those retailers take additional measures to ensure the quality of the products. Products were vacuum sealed and contained moisture packets. As one participant said: “*they go the extra mile… to make sure that your product is…almost as it was, when it was cured and cut*”.

### Social influences

Several social influences were discussed with respect to purchase decisions, including product recommendations, stigma, and personal safety. While some participants were knowledgeable and knew exactly what product they wanted to buy, those with less experience took product recommendations from store staff or friends into consideration. As one person said, “*I go almost solely off recommendation when I go to legal stores.*” Some participants were also interested in hearing recommendations about new product types or strains. For individuals who shopped through unlicensed sources online, they used online product reviews to ensure that both the product and the business had a good reputation for quality and service. With regard to medical use, most people indicated that they would not expect to get any medical advice from a cannabis retailer. However, participants indicated that they would like to get information and recommendations based on a desired effect or experience (e.g. relaxation, stimulation, sleep, anxiety, pain, etc.).

Most of the participants in this study were experienced users and were not concerned about stigma when purchasing cannabis. That being said, several participants highlighted how stigma could potentially influence or discourage cannabis purchasing decisions for other people. There are cannabis retailers located in high-traffic areas or areas with restaurants with outdoor seating nearby. One person said, “*when I walk into the [cannabis store] door, everyone just gawks over looking at you.*” This kind of social behaviour may be intimidating to some customers.

Most participants felt that there was a general shift in how people perceive cannabis, with less stigma than before. One participant noted that the stigma has not been eliminated: “*I think although it might not be as expressed as much, it’s still there*.” Negative stereotypes still exist, especially among the older demographic. Some participants discussed how they were not as quick to reveal their cannabis use in front of colleagues as they would be for alcohol, as they were unsure of how they would be perceived. The degree of stigma in the community also depended on the location. Those who had experience purchasing cannabis in other provinces noted that NL tended to be more progressive and accepting of cannabis than many other provinces. As one person said, “*It’s incredible if you ever leave the province and try to buy cannabis somewhere else, how backwards and unprogressive they are with it*.”

The issue of personal safety was also raised. This was not a common concern, but one woman indicated that personal safety was her number one priority. She preferred to purchase from a store and did not want to be in a situation where she had to meet someone alone. She said, *“I like… the cannabis stores… because it’s really accessible. And… it’s really open and I feel safe again.”*

### Retailer characteristics

Many participants commented on retailer characteristics that impacted choice. These included product availability and variety (including products from craft cultivators), location proximity, hours of operation, and the knowledge of staff. One person also discussed how supporting local, legal businesses was a critical factor influencing their purchase decisions. “*The biggest thing for me is that it’s a local company... I want to make sure that my money is staying in the province. So where I go is dependent on whether it is locally run or not.*”

The availability and variety of products from particular sources was an important factor in participants’ choices of where to make their purchase. The participants felt, however, that many of the supply and variety issues had been resolved since production ramped up and edibles were legalized for sale.

Product variety issues remained, primarily with cannabis concentrates and edibles. Some participants suggested they would like to see concentrates available, but they have not yet been approved for the legal market. While edibles have been approved, the maximum dose of tetrahydrocannabinol (THC) per package is set to 10 mg, which was a concern for edible consumers. Those with experience suggested that “*you gotta buy like four or five packages, just to, you know to get a buzz going on edibles*”, and they ended up purchasing from unlicensed sources to get higher-strength products.

Additionally, smaller stores (often in rural areas) could not offer the same variety of products than those offered in larger stores in urban centres. Because there is not a big enough market for each product type, they can only stock more common product types. This was noted to be problematic for people who preferred lower-strength THC products and/or higher-strength cannabidiol (CBD) products, which were not always stocked in smaller stores.

The participants did not want to travel far to get what they were looking for, and they wanted to get it at a time that worked for their needs. As one person said: *“I just … go to the local place, whichever is closest.”* Several people noted that some legal stores have limited hours of operation and close too early; however, when speaking about unlicensed neighborhood retailers, one person said: *“when you get somebody … they’re open almost 24 hours, whereas when you go to a store you would have to go during set hours.”* It was also noted that unlicensed retailers, particularly neighborhood retailers, have adapted to the new legal market to remain competitive. This included being available for more hours and providing convenience services. One person mentioned: “*you can get yourself door-delivery of cannabis, no problem at a pretty good price.”*

Finally, the knowledge and openness of the staff were discussed. These comments were more focused on the presence of helpful and courteous staff. There was reference to certain stores where staff not only lacked a level of awareness about the products but also gave customers a sense of being judged for the purchases they were making. In contrast, other stores make people feel very welcomed and respected, improving their overall experience.

## Discussion

This study was the first qualitative study to engage with consumers to understand their perspectives on the factors that were relevant and important when purchasing a cannabis product. Price appeared to be the most important factor that influenced cannabis purchase decisions; however, there were many other factors that influenced participants’ choice of product, including quality, packaging, social influences, and retailer characteristics.

The findings of this study are in line with multi-attribute utility theory, which states that when people make choices they take into consideration the various elements of that choice [13]. They then make trade-offs between the elements that perform less than ideally (or poorly) and elements that perform well. In our study, while price was important, many people were willing to pay more for products that were of a superior quality, recommended, or had value-added customer service. A discrete choice experiment was conducted in areas of the United States where cannabis was legal to measure the trade-offs people consider when purchasing cannabis products [14]**.** While this study only explored a narrow set of attributes, it demonstrated that cannabis consumers would pay more for cannabis flower that contained a higher concentration of CBD or THC. That study also demonstrated differences in preferences between people who consumed cannabis for medical or non-medical purposes. Further research is needed to assess trade-offs with a wider range of attributes of choice. A better understanding of the trade-offs made by cannabis consumers in Canada would allow for decision makers to identify specific policies and regulations that may influence purchase behaviours. This can inform refinement of specific regulations (e.g. cultivation processes, packaging, price) to nudge consumers towards licensed retailers, while still protecting public health and safety.

Our study suggested that purchase choices differed based on the characteristics of the consumer. While it was not possible to differentiate preferences between use for medical or non-medical purposes, gender or age, there were some obvious differences between those who were experienced and those who were less experienced. Those with experience were more concerned about price, partly because they tended to consume more cannabis and were already comfortable with purchasing through unlicensed sources. Perceived quality was also different based upon level of experience. Less experienced consumers focused more on labelling and product testing, while those with more experience considered product elements like the curing process, terpenes, trichomes, and moisture content. The potency or strength of the CBD or THC was relevant to people of all levels of experience; however, preferences for higher strengths of THC were more common for experienced users. Preferences towards smell and visual properties and higher THC strength among more experienced users were supported by previous research [15].

The distinction in perceived quality was important to note, as there appeared to be a lack of a formal definition for cannabis quality both in the literature and within the licensed market. Several studies [16–20] explored the impact of quality on choices, but this term has not been defined in relation to cannabis. Knowing that quality can mean different things to different people means that previous studies tell us little about what specific elements of cannabis quality are important in guiding consumer choices. The unlicensed market often uses the AAAA grading system. While there is no oversight body that provides objective criteria for this grading system, it is commonly referenced on cannabis blogs, social media, and unlicensed retailer websites. One such blog [21] indicated that a final grade is based on a combination of the flower structure, trichome density, trim, terpene profile, how it burns, colour of the ash, flavor, effect, and use of sprays or pesticides. This highlights the need for objective measurement criteria to use to assess cannabis quality within the legalized cannabis industry. This would not only be beneficial for consumers but also for cultivators, retailers, and researchers.

There were several limitations in conducting this study. First, the most common method of use by participants was smoking dried cannabis flower, so we were unable to fully understand purchase decisions for other product types (e.g., edibles, beverages, oils, concentrates). While the Canadian government legalized edibles and topicals in October 2019, it was not until the spring of 2020 before individual products were approved and available in stores. Therefore, moving forward we may see a rise in popularity of other product types, making it easier to explore purchase preferences. Secondly, there is a large disparity between geographic areas of the province of NL when it comes to access to cannabis retailers, both legal and illegal. While we had representation from all four regions of the province and from rural and urban communities, there may be factors that were not fully explored based upon geographical location.

Based on these qualitative findings, it is not possible to determine the relative importance of the attributes identified. It is not possible to say that, for example, quality is more important than location, or vice versa. We can only describe the attributes that consumers describe and consider relevant. With further research in the area of consumer preferences, we can gain a deeper understanding of the factors that influence purchase decisions. This knowledge could be used to support cannabis policy and regulation changes to help better align them with consumer needs and ultimately make the legal cannabis market more appealing to those who purchase cannabis.

## Conclusion

Purchase decisions for cannabis products involve the consideration of multiple factors. Like most other purchase decisions, price appears to be the most important consideration; however, people do make trade-offs with price in return for other aspects such as higher quality, product recommendations from peers and/or retailers, improved customer support, and convenience. While this study provided some insight into purchase decisions, further research is needed to assess the relative importance of the characteristics that influence choice, to distinguish preferences among population sub-groups, and to distinguish preferences between different cannabis product types. A more detailed understanding of purchasing behaviour can support policy refinement to better support consumer preferences.

## Data Availability

The datasets generated and/or analysed during the current study are not publicly available, as they may lead to the identification of participants, but they are available from the corresponding author on reasonable request.

## Abbreviations

CBD: Cannabidiol
ICEHR: Interdisciplinary Committee on Ethics in Human Research
NL: Newfoundland and Labrador
THC: Tetrahydrocannabinol
